# Coxsackievirus B3, Shandong Province, China, 1990–2010

**DOI:** 10.3201/eid1811.120090

**Published:** 2012-11

**Authors:** Zexin Tao, Yanyan Song, Yan Li, Yao Liu, Ping Jiang, Xiaojuan Lin, Guifang Liu, Lizhi Song, Haiyan Wang, Aiqiang Xu

**Affiliations:** Shandong Center for Disease Control and Prevention, Jinan, People’s Republic of China (Z. Tao, Y. Li, Y. Liu, X. Lin, G. Liu, L. Song, H. Wang, A. Xu);; Shandong University School of Public Health, Jinan (Y. Song, A. Xu);; and Stony Brook University, Stony Brook, New York, USA (P. Jiang)

**Keywords:** coxsackievirus B3, aseptic meningitis, acute flaccid paralysis, phylogenetic analysis, viruses, China

## Abstract

To determine the cause of a 2008 outbreak of aseptic meningitis in Shandong Province, China, we analyzed samples from outbreak patients and coxsackievirus B3 samples collected during 1990–2010 surveillance. The cause of the outbreak was coxsackievirus B3, genogroup D. Frequent travel might increase importation of other coxsackievirus B3 genogroups.

Coxsackievirus B3 (CVB3) (family *Picornaviridae,* genus *Enterovirus*) is a major human pathogen (*1*–*3*), and CVB3-associated aseptic meningitis is an emerging concern (*4*). In the summer of 2008, an aseptic meningitis outbreak occurred in southern Shandong Province, People’s Republic of China. Shandong is a coastal province with a population of 94.7 million. The huge number of hospitalized children caught the attention of public health officials and media and triggered an extensive study on the causative agent. To help determine the cause of the outbreak, we analyzed samples from outbreak case-patients and conducted a molecular epidemiology study of CVB3 isolates collected in Shandong during 1990–2010.

## The Study

The 2008 outbreak occurred during June–September and peaked in early July. A total of 887 patients, 596 male and 291 female, were hospitalized. Patient ages ranged from 2 months to 64 years; most (98%) were <15 years of age. Epidemiologic investigation showed that 617 (69.6%) case-patients had a history of contact with aseptic meningitis case-patients, and 159 (17.9%) had drunk untreated well water. The most common clinical manifestations were fever (89.6%), vomiting (57.2%), headache (48.3%), lethargy (14.5%), and rash (1.5%). No sequelae or deaths were reported.

To investigate the causative agent, we collected 120 cerebrospinal fluid samples and 22 fecal samples from 142 case-patients. Using the RD and HEp-2 cell lines, we isolated 82 enteroviruses (67 from cerebrospinal fluid and 15 from feces); serotypes were CVB3 (n = 81) and echovirus 30 (n = 1). We sequenced and analyzed the virus capsid protein 1 (VP1) regions of 34 randomly selected CVB3 strains as described (*5*–*7*). Only strain TC177 had 8.1%–9.7% genetic divergence with the other 33 strains, which shared 98.6%–100.0% identity among themselves.

We also analyzed details of clinical and environmental CVB3 strains collected in Shandong since 1990 (Table) by using data from the acute flaccid paralysis (AFP) surveillance system. Because China has no specialized enterovirus surveillance system, no comprehensive molecular epidemiologic data are available for CVB3 or other nonpolio enteroviruses that cause aseptic meningitis. However, the AFP surveillance system, developed for the polio eradication program and conducted in Shandong since 1990, can provide baseline data of local nonpolio enterovirus circulation (*8*).

**Table:** Sources of 157 coxsackievirus B3 in Shandong Province, People’s Republic of China, 1990–2010*

Source	Specimen	No. isolates	Years of isolation
AFP surveillance	Feces	59	1990–2010
Environmental surveillance	Sewage	9	2008–2010
Patients with aseptic meningitis and encephalitis syndrome in Jinan	CSF	4	2006–2008
Patients with aseptic meningitis during outbreak in Tancheng	CSF and feces	81	2008
Patients with aseptic meningitis during outbreak in Yanzhou	CSF	2	2005
Patient with hand-foot-and-mouth disease	Throat swab	2	2008

*AFP, acute flaccid paralysis; CSF, cerebrospinal fluid.

**Figure 1:**
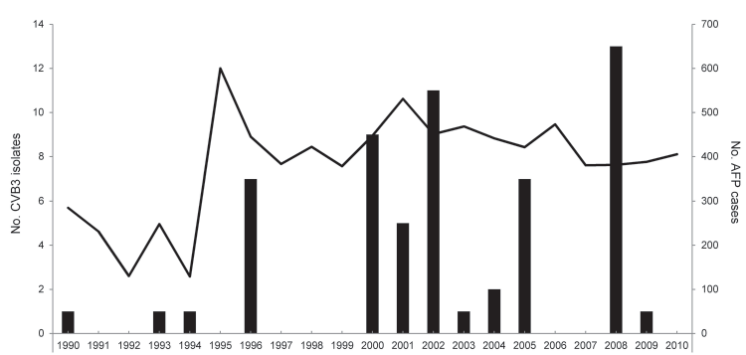
Frequency of isolation of coxsackievirus B3 (CVB3) in patients with acute flaccid paralysis, Shandong Province, People’s Republic of China, 1990–2010. Bars indicate number of CVB3 isolates from acute flaccid paralysis (AFP) surveillance; line indicates number of cases of AFP.

According to the AFP surveillance system, during 1990–2010, a total of 768 nonpolio enteroviruses were isolated, and 59 (7.7%) strains were typed as CVB3 by use of a molecular method (*6*). Frequency of CVB3 isolation fluctuated from year to year and peaked during 1996, 2000–2002, 2005, and 2008 (Figure 1). The low level of CVB3 before 1996 might be a surveillance artifact because AFP surveillance in China increased substantially throughout the 1990s. CVB3 accounted for ≈21% of all nonpolio enterovirus isolates from AFP case-patients in 2008. Another outbreak of aseptic meningitis outbreak occurred in 2005, during which 57 CVB5 strains were recovered (*9*) and 2 CVB3 strains were identified. From environmental surveillance, CVB3 was isolated in 2008 (n = 7), 2009 (n = 1), and 2010 (n = 1). In addition, CVB3 was isolated from patients with hand-foot-and-mouth disease (n = 2) and sporadic aseptic meningitis and encephalitis syndrome (n = 4).

**Figure 2:**
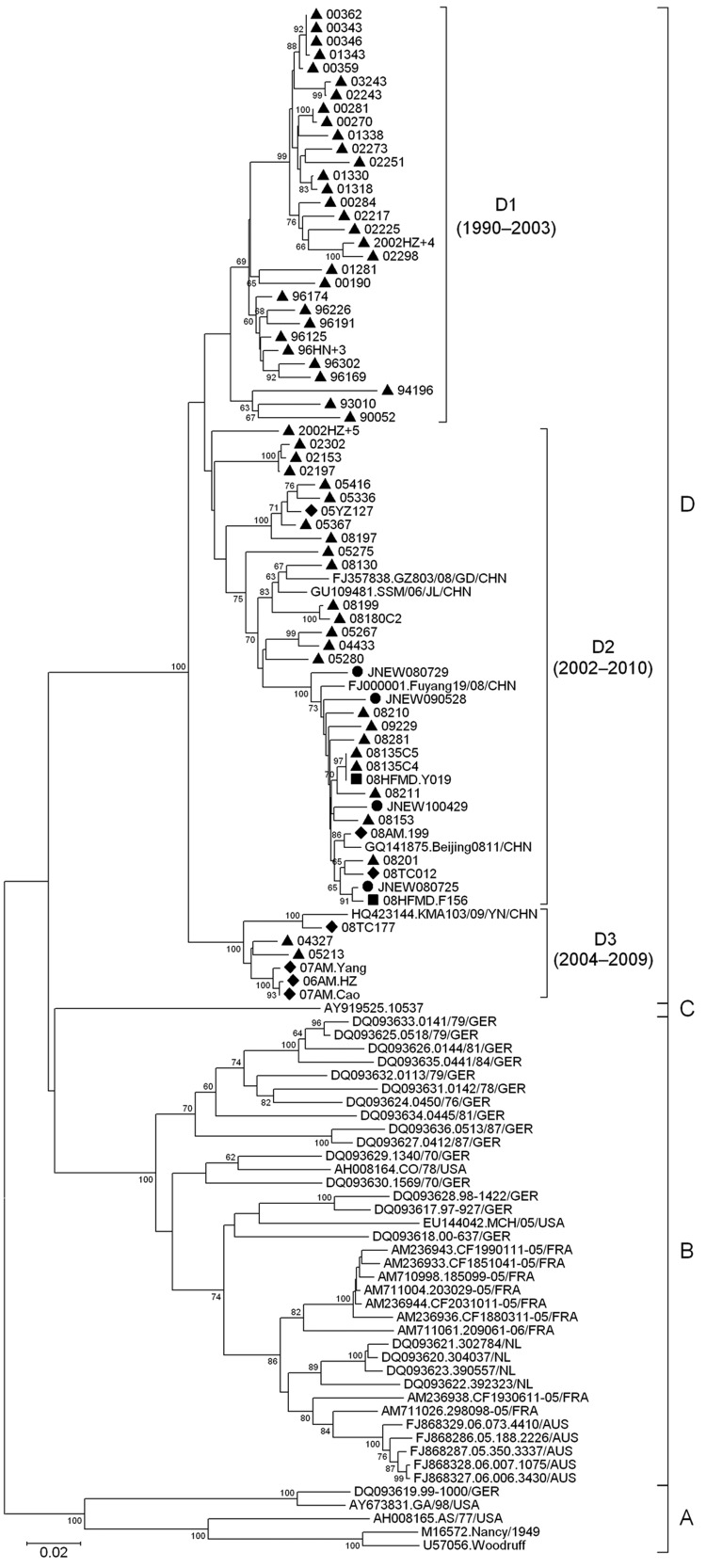
Phylogenetic tree based on the alignment of the entire virus capsid protein 1 coding regions of coxsackievirus B3 isolates from Shandong, People’s Republic of China, and around the world. Triangles indicate isolates from patients with acute flaccid paralysis; diamonds indicate isolates from patients with aseptic meningitis; circles indicate isolates from the Shandong environment; and squares indicate isolates from patients with hand-foot-and-mouth disease; the arrow indicates the representative strain 2008TC012 from the aseptic meningitis outbreak in 2008. Strain 26362/08 from patients with aseptic meningitis in Hong Kong is not shown in the tree because only part of the virus capsid protein 1 sequence was available. AUS, Australia; CHN, China; FRA, France; GER, Germany; NL, the Netherlands; USA, United States. Scale bar indicates nucleotide substitutions per site.

To study the molecular epidemiology of CVB3 in Shandong, we used MEGA version 4.0 (*10*) to phylogenetically analyze the complete VP1 sequences of global CVB3 strains, including 72 Shandong isolates from AFP surveillance (n = 59), aseptic meningitis case-patients (n = 7), hand-foot-and-mouth disease case-patients (n = 2), and environmental surveillance (n = 4) (Figure 2). Global CVB3 strains were segregated into 4 genogroups, A–D, with at least 15% complete VP1 nucleotide diversity between clusters. All strains from Shandong, together with 5 strains from the Chinese provinces of Guangdong, Jilin, Anhui, Yunnan, and Beijing, fell into genogroup D, which was further divided into 3 subgenogroups, D1–D3, representing isolates from 1990–2003, 2002–2010, and 2004–2009, respectively. Homologous analysis indicated that nucleotide identities among subgenogroups D1–D3 were 90.9%–100%, 89.6%–100%, and 94.4%–99.6%, respectively. The representative strain from the aseptic meningitis outbreak (TC012) belonged to subgenogroup D2, and TC177 belonged to subgenogroup D3.

When partial VP1 sequences (393 nt) were aligned, the relationship between Shandong strains in 2008 and Hong Kong aseptic meningitis strain 26362/08 was close: strains 08153 and 08281 had the highest similarities (98.7%) with 26362/08. A close relationship (97.7%–98.5% similarity) was also observed between 26362/08 and the other aseptic meningitis strains from Shandong (TC047, CVB3 strain 08AM.199, hand-foot-and-mouth disease strain Y019, environmental stains in 2008, and the strains from Beijing, Anhui, and Yunnan from 2008).

The VP1 sequences were deposited into GenBank. Accession numbers are GQ246518, GQ329744–GQ329767, FJ919564, FJ919566–FJ919598, GU272011–GU272013, and JQ364844–JQ364885.

## Conclusions

Our results indicate that CVB3 was the cause of the outbreak and that most CVB3 isolates were closely related. The AFP surveillance in Shandong, although insufficient for monitoring enterovirus infections of humans, revealed a similar fluctuating epidemic mode for CVB3 with a temporal peak in 1–3 years and quiescence for 2–3 years. The temporal dynamics of echovirus 30, causing nationwide epidemics of 2–4 years separated by periods of quiescence, have been reported (*11*). The CVB3 peak detected by AFP and environmental surveillance in 2008 correlated well with the aseptic meningitis outbreak, demonstrating high CVB3 activity at that time. Of note, aseptic meningitis surveillance in Hong Kong detected increased CVB3 activity during 2001, 2005, and 2008 (*4*), consistent with the temporal peak of CVB3 in Shandong. The close genetic relationship between the strains from mainland China and the Hong Kong strains in 2008 suggests that the same strain was circulating in both regions.

Most global CVB3 strains belong to genogroups B and D. During 1970–2006, genogroup B came from the United States, Australia and Europe. However, this genogroup has not yet been found in mainland China. Although genogroup D was composed entirely of strains from China, our data are insufficient for us to propose that genogroup D is confined to China only. Nevertheless, our study demonstrates that genogroup D has been predominantly circulating in mainland China for the past 20 years and is responsible for all documented outbreaks and sporadic cases of CVB3-associated aseptic meningitis. Because of the more frequent population exchange between China and the rest of the world, the chance for importation of other CVB3 genogroups to mainland China is greatly increased.

## References

[1] Khetsuriani N, Lamonte-Fowlkes A, Oberste S, Pallansch MA. Enterovirus surveillance—United States, 1970–2005. MMWR Surveill Summ. 2006;55:1–20. 16971890

[2] Baboonian C, Davies MJ, Booth JC, McKenna WJ. Coxsackie B viruses and human heart disease. Curr Top Microbiol Immunol. 1997;223:31–52. 10.1007/978-3-642-60687-8_3 9294924

[3] Hyöty H, Taylor KW. The role of viruses in human diabetes. Diabetologia. 2002;45:1353–61. 10.1007/s00125-002-0852-3 12378375

[4] Wong AH, Lau CS, Cheng PKC, Ng AYY, Lim WWL. Coxsackievirus B3–associated aseptic meningitis: an emerging infection in Hong Kong. J Med Virol. 2011;83:483–9. 10.1002/jmv.21998 21264869

[5] Tao Z, Wang H, Li Y, Xu A, Zhang Y, Song L, Cocirculation of two transmission lineages of echovirus 6 in Jinan, China, as revealed by environmental surveillance and sequence analysis. Appl Environ Microbiol. 2011;77:3786–92. 10.1128/AEM.03044-10 21478313 PMC3127621

[6] Oberste MS, Maher K, Kilpatrick DR, Pallansch MA. Molecular evolution of the human enteroviruses: correlation of serotype with VP1 sequence and application to picornavirus classification. J Virol. 1999;73:1941–8. 9971773 10.1128/jvi.73.3.1941-1948.1999 PMC104435

[7] Oberste MS, Maher K, Kilpatrick DR, Flemister MR, Brown BA, Pallansch MA. Typing of human enteroviruses by partial sequencing of VP1. J Clin Microbiol. 1999;37:1288–93. 10203472 10.1128/jcm.37.5.1288-1293.1999 PMC84754

[8] Bingjun T, Yoshida H, Yan W, Lin L, Tsuji T, Shimizu H, Molecular typing and epidemiology of non-polio enteroviruses isolated from Yunnan Province, the People’s Republic of China. J Med Virol. 2008;80:670–9. 10.1002/jmv.21122 18297723

[9] Wang HY, Li Y, Xu AQ, Zhang Y, Tao ZX, Liu GF, Identification and phylogenic analysis of coxsackievirus B5 during an outbreak of aseptic meningitis in Shandong [in Chinese]. Zhonghua Liu Xing Bing Xue Za Zhi. 2010;31:64–8. 20302702

[10] Tamura K, Dudley J, Nei M, Kumar S. MEGA4: Molecular Evolutionary Genetics Analysis (MEGA) software version 4.0. Mol Biol Evol. 2007;24:1596–9. 10.1093/molbev/msm092 17488738

[11] Oberste MS, Maher K, Kennett ML, Campbell JJ, Carpender MS, Schnurr D, Molecular epidemiology and genetic diversity of echovirus type 30 (E30): genotypes correlate with temporal dynamics of E30 isolation. J Clin Microbiol. 1999;37:3928–33. 10565909 10.1128/jcm.37.12.3928-3933.1999 PMC85848

